# Thinking Big or Small: Does Mental Abstraction Affect Social Network Organization?

**DOI:** 10.1371/journal.pone.0147325

**Published:** 2016-01-25

**Authors:** Chantal Bacev-Giles, Johanna Peetz

**Affiliations:** Department of Psychology, Carleton University, Ottawa, Ontario, Canada; Universidad de Alicante, ITALY

## Abstract

Four studies examined how mental abstraction affects how people perceive their relationships with other people, specifically, how these relationships may be categorized in social groups. We expected that individuals induced to think abstractly would report fewer more global social groups, compared to those induced to think concretely, who would report more specific groups. However, induced abstract mindset did not affect how people structured their social groups (Study 2–4), despite evidence that the mindset manipulation changed the level of abstraction in their thoughts (Study 3) and evidence that it changed how people structured groups for a control condition (household objects, Study 4). Together, these studies suggest that while the way people organize their relationships into groups is malleable; cognitive abstraction does not seem to affect how people categorize their relationships into social groups.

## Introduction

On a daily basis individuals encounter and interact with many different people. Some of these people may be strangers or slight acquaintances; others may be family, friends, and colleagues–a variety of social relationships. Individuals tend to organize and impose structure across many domains of life [1], one of which may include the social world. This research explores how people structure their social network and examines whether the level of abstraction in their frame of mind may affect the level of abstraction they use to form these structures.

Understanding how individuals structure their social networks can provide a framework for understanding interpersonal relationships and one’s behavior within those relationships. For instance, Jessica may imagine her social world to consist of very few and superordinate social groups such as: family, friends, and colleagues. Matthew on the other hand may perceive his social world to consist of many specific groups such as: immediate family members, school friends, and sports friends. Structure may be important for relationships as individuals like Matthew might have different relational experiences and a more differentiated identity [2,3] than someone like Jessica who integrates her relationships more. Attesting to the importance of social structures, people spontaneously engage in a variety of social networking sites (e.g., Facebook, Google+) which help sort and categorize their different relationships. For example, Facebook prompts its users to sort contacts into “close friends” versus “acquaintances” while also allowing users to create names for other personalized categories. Social media plays an active role in people’s daily routines [4], and with its increased use and applications for information and communication, the number of ways people can interact with and organize their various relationships is limitless.

The perceptions and interactions individuals have with these various people may change by virtue of how they structure their social groups. Reframing one's social network in different groups (e.g., global groups such as "family" versus more specific groups differentiating "in-laws" from "household family") may affect the importance these groups have for identity [3]. Furthermore, the valence and emotional meaning of the groups may shift alongside the relationships that are included in them. Attitudes towards more global, abstract social groups might be diluted by the variety of relationships included in them whereas people might feel more emotionally connected and close to more specific, concrete groups. Similarly, failing to cognitively separate some relationships into different social groups could lead to conflict: work-family conflict has been linked to a lack of boundaries between work and family domains [5]. Thus, structuring a network in many specific social groups might be linked to greater distinctiveness of each group, which can help people prevent emotional spill over. In sum, the way social groups are structured can have important consequences.

### Mapping Social Groups

How do individuals classify their relationships into social groups? Despite many attempts to map the social world [6], there is no consensus on how individuals structure their social networks. Definite classifications of the social world remain elusive [6,7]. One reason for this is that there is an abundance of terms and definitions related to social groups that are often used interchangeably. For instance, the term “social group” which defines a specific group one identifies with has also been used interchangeably with: social world [8], social networks [6,7,9], social bonds, social support, primary relationships (e.g., family and friends), and informal groups [10]. For the purpose of this research we used the term *social group* to represent a cluster of people that an individual knows and frequently interacts with, who are interconnected, and are grouped on the basis of specific behavioral and affective distinctions [7,11]. The number of social groups varies from person to person and over the life cycle [10,12].

In a review, Milardo [6] states that there are four common types of social groups: significant others (e.g., family, close friends, romantic partner), exchange networks (e.g., people who provide support and resources; friends, neighbors, colleagues), interactive networks (e.g., people one regularly interacts with, does not necessarily overlap with the other groups), and the global network (e.g., entire social network). While this provides a solid theoretical base for social network classification, one disadvantage is that other studies have failed to consistently document similar classifications or even agree on the group size for these social groups. One study noted that individuals had an average of 26 interactive network members and interacted with an average of 4.6 of these members per day [13]. In another study, Milardo [14] noted 16 interactive network members and that people interact with an average of 2.4 of these people per day. Asendorpf and Wilpers [15] note that young adults maintain an overall average of 37 meaningful social relationships. These conflicting reports may be reconciled by considering the cognitive mindset adopted in the moment which may shift the way social groups are structured.

### Shifting Social Group Structure

A number of factors may shift social network size [16], including demographic variables such as relationship status [11,13,17], gender [18,19,20], age [18,19], education [12,18] and personality variables such as extraversion [18,19] and neuroticism [21].

Demographic variables such as relationship status matter: single or recently dating individuals tend to have more interpersonal relationships than individuals who have been dating for longer periods of time or are engaged [13,17]. Interactions with others during the marital stage are predominately with one type of social group (e.g., kin), whereas after a separation one’s network size begins to resemble the premarital network, characterized by longer-lasting interactions with other types of social groups (e.g., friends) and fewer interactions with kin [11]. Personality variables such as extraversion predict the size of some social groups (e.g., friends) but not others (e.g., family [18]). Another study suggested that extraversion is correlated to the size of one’s support groups (e.g., best friends and intimates) but not correlated to the size of one’s sympathy groups (e.g., one’s principle group of friends [17]).

In the present research, we examine not social networks or social group *size* but the *number* of social groups individuals chose to sort their various relationships into. While demographic and personality variables have been shown to change social network size or the size of individual groups, the way this overall network is structured into groups remains elusive. The network may be conceived of in abstract ways (sorting relevant relationships into global, abstract categories) or in very concrete ways (sorting relevant relationships into specific, concrete categories. We examine one variable that might affect the way people structure their social groups (i.e., the number of social groups they use to categorize the network): level of abstraction in thought. Specifically, we examine whether thinking abstractly can shift individuals' social network structure. Does thinking abstractly lead to constructing more abstract, global social groups, and thinking concretely lead to more concrete, specific social groups? Frame of mind was operationalized as construal level mindset.

### Construal Level Mindset

Construal Level Theory [22,23] suggests that people’s thoughts can shift in the degree of abstraction, depending on an individual's predisposition or situational cues. Abstract thought captures the global, primary features of an event, compared to concrete thought which captures the specific details or secondary features of an event [23,24]. For example, someone who considers the action of reading a paper may focus on specific, concrete aspects of this action (e.g., following words on a page) rather than focusing on the purpose of the action (i.e., learning something), depending on their construal mindset. In turn, asking people to focus on the purpose of a specific action versus the process of a specific action can shift the abstraction of people's mindset and change their interpretations of later events [25]. In the social realm, the abstraction of thought (i.e., construal mindset) can change how ambiguous social situations are interpreted: when participants were in a concrete construal mindset, they interpreted an ambiguous social situation (a first date) as likely leading to a one-night stand (i.e., a specific, short term action) whereas when participants were in an abstract construal mindset, they interpreted the same situation as leading to a long-term commitment [26]. In turn, asking people to focus on sex (concrete action) versus love (abstract purpose) can shift construal mindset and change partner perception [27], and perception more generally [28].

In the past twenty years, the level of abstraction in thought, as operationalized via construal level mindset, has been shown to pervade almost any aspect of individuals' lives [23], including preferences [29], decisions [30], and behaviors such as procrastination [31], and self-control [32]. Most pertinently, Liberman and colleagues [33] showed that the mental categorization of objects can change based on people's level of abstraction in thought. Participants who adopted a concrete mindset when thinking about an event (e.g., moving out) constructed more specific categories to classify the objects connected to the event. Participants who adopted an abstract mindset when thinking about the same event sorted the objects into fewer, more global categories. Thus the degree of abstraction present in people's frame of mind at a given moment plays an important role in how they think about the world.

Given the pervasive impact of the level of construal on everyday thoughts and behaviors [22–25,33] including the organization and classification of objects [33], it is possible that construal mindset also affects how we categorize and classify one of the most important aspects of our world: social relationships. An individual who adopts an abstract mindset may categorize their social relationships into fewer, more global categories than someone who adopts a concrete mindset.

### Overview

The purpose of the current research is to explore individuals' social groups and whether the inclusiveness of social groups can be shifted by the level of abstraction in their mindset that prevails at a given moment. Given the wide variety of documented social groups that individuals divide their network into [13–20], groups might shift depending on context and cognitive mindset in any given moment, so that the structure imposed on one's social network is in flux. The level of abstraction in thought is one of the most fundamental principles in social cognition that can change one’s outlook on the world. As such, it might be the most powerful test of a situational or mindset-dependent shift in how social networks are construed.

Study 1 examines individuals' 'typical' number of social groups, when construal mindset is left free to vary. In Studies 2 to 4, we assign individuals to adopt an abstract construal mindset or a concrete construal mindset and examine this effect on the number of their social groups. We expect that inducing an abstract mindset will lead people to shift their social groups to include fewer, more global categories than a concrete mindset. Study 4 investigated the effect of construal priming not only for social groups but also for a comparison domain–objects. We expect that similar to past findings [33], individuals will sort objects into fewer, more global categories when adopting an abstract thought mindset than when adopting a concrete thought mindset.

### Ethics Statement

Ethics approval for all studies were obtained from the Carleton University Psychology Research Ethics Board. In Study 1, Study 2, and Study 4, an online questionnaire was employed in which participants provided their consent by selecting an “agree” or “disagree” (i.e., exit survey). For Studies 3 and 4 in which a physical survey was administered, participants provided written consent.

## Study 1

This study explored the number of social groups individuals tend to construct from their relationships as well as the labels they attach to these groups. Importantly, the number of social groups should be independent of the overall network size (whether someone's family consists from 13 or three members, two people might equally chose to apply the label of 'family' to this aspect of their social network). In this initial study we also explored the potential effects of choosing global versus specific social groups on attitudes and feelings towards these groups.

### Method

#### Participants

We recruited 85 participants from the online crowd-sourcing website CrowdFlower to complete a survey on social groups. Participants received $0.50 US as compensation for the 5-minute survey. Three participants did not complete all measures and were excluded and an additional six were excluded for providing nonsensical responses in the social group measure. The final sample consisted of 76 participants (29 females and 47 males, *M*_age_ = 35.30, *SD* = 13.14).

#### Procedure

Participants first read a generic definition of s*ocial group*s: “A social group is defined as a group of people sharing something in common”. To avoid priming participants with a particular level of inclusiveness or globality, this definition did not provide any examples. Participants were asked to list their social groups in an open textbox, separated by semicolons. A single textbox was used to prevent cuing participants to an expected number of social groups. Next, participants reported how many people they tend to interact with on a daily and monthly basis. Two values were excluded in analyses of these variables as they were outliers (more than three standard deviations above the mean).

To assess potential effects of categorizing one's social relationships into few versus many social groups, we also assessed two related attitudes: perceived distinctiveness of the social groups and felt closeness to the social groups. Three items assessed group distinctiveness (“It is important for me to maintain many different social groups.”, “I prefer to keep my social groups separate from each other.”, and “My social groups are quite distinct from one another.”) and 3 items assessed group closeness (“I feel connected to others.”, “My social groups form a part of my identity”, and “I have a sense of closeness and connection with my social groups.”). All items were anchored on a scale from 1 (*strongly disagree*) to 5 (*strongly agree*). Aggregate scores were computed for these two dimensions, where high scores represented greater distinctiveness between social groups, and greater closeness to one’s social groups, respectively. Finally, participants completed a demographics survey reporting only age, gender, and relationship status.

### Results and Discussion

On average, participants listed 3.24 (*SD* = 1.49) social groups (normally distributed, with 6 participants listing one group, 21 listing two groups, 21 listing three groups, 15 listing four groups, six listing five groups, five listing six groups, and one participant listing seven and eight groups, respectively). The number of social groups did not differ by gender, *t*(74) = -1.14, *p* = .260, *d* = -0.25, and did not correlate with age, *r*(76) = .09, *p* = .455. The number of social groups did not differ for people who were single (*n* = 31) versus in a steady relationship or married (*n* = 45), *t*(74) = .10, *p* = .919, *d* = 0.02.

People reported interacting with an average of 958. (*SD* = 18.94) people on a daily basis and 62.22 (*SD* = 179.32) people on a monthly basis. The number of social groups was uncorrelated to daily interactions (*r*(71) = -.06, *p* = .595) and monthly interactions (*r*(71) = -.10, *p* = .419). This suggests that the way people chose to categorize their social relationships is independent of people’s overall social network size.

Most frequently mentioned were three main social group categories: friends (as general category: 69.7%, where all participants mentioned at least one type of friend, such as a "school friend"), family (as general category: 36.8%, all participants mentioned at least one type of family, such as "immediate family"), and colleagues (mentioned by 21.1% of the sample). This finding is consistent with the three types of social groups mentioned by Milardo [4,12]. Attesting to the importance of social media, 28.9% of participants mentioned social media platforms as a type of social group at least once, suggesting that perhaps social media is an important component to identifying with one’s relationships and categorizing them into groups. Furthermore, some participants distinguished between more specific friend groups (40.8%) such as activity friends (generalized across various specific extracurricular activity groups), school friends, best friends, and social networking friends (14.3%). A few listed even finer distinctions for their family members such as extended family and immediate family. In addition, 7.9% of participants mentioned neighbors and acquaintances. Any other listed social groups, including those which were not recognizable groups (e.g., social groups distinguished by abbreviations or acronyms) were categorized as “others”' (26.3%).

Next we examined potential effects of the number of social groups for participants' feelings and attitudes. The number of social groups was linked with perceived social group distinctiveness, *r*(74) = .24, *p* = .041, suggesting that fewer, more global groups were less distinct from each other than a greater number of more specific groups. In turn, the more distinct the social groups were from each other, the closer participants reported feeling to their social groups, *r*(74) = .35, *p* = .002. the number of social groups was not correlated with closeness, *r*(41) = .10, *p* = .406, however the indirect effect of the number of social groups on group closeness, via group distinctiveness, was significant (*M* = 0.04, *SE* = 0.03, 95% CI [0.01, 0.12]).

In sum, some participants listed more abstract categories such as “friends”, whereas others listed more specific categories such as “school friends” and “travel friends”. It could be that participants view their social groups through varying levels of abstraction (i.e., concrete versus abstract mindsets). The next study examines whether shifts in mindset can shift people's number of social groups.

## Study 2

Is abstraction in thought linked to abstraction in the categorization of social groups? We predicted that participants who adopted an abstract mindset would report fewer, more abstract social groups (e.g., “friends”) compared to those who adopted a concrete mindset who would report many specific social groups (e.g., “best friends”, “school friends”, and “activity friends”).

### Method

#### Participants

We recruited 153 American participants from the website CrowdFlower to participate in an online study. Participants received $0.50 US as compensation. Twelve participants were excluded because they did not complete the manipulation, and 11 were excluded for not completing the social group measure (e.g., providing nonsensical responses). The final sample included 130 participants (65 females, 65 males) with a mean age of 35.22 years (*SD* = 12.15).

#### Procedure

Participants were randomly assigned to one of two conditions: an abstract mindset or a concrete mindset condition [25,32]. Participants in the abstract construal mindset condition were asked “why” they would maintain a goal (health improvement), and participants in the concrete construal mindset condition were asked “how” they would maintain this goal. Using four textboxes which were connected by arrows, participants thought about four increasingly abstract, higher level thoughts for why they would maintain this goal or four increasingly concrete, lower level thoughts for how they would maintain this goal. Accordingly, all participants focused on the same goal while varying only the abstraction level with which they thought about this goal. This manipulation operates on the principle that the level of abstraction induced by this thought exercise then remains active in people's minds.

Next, using the same definition of social groups as in Study 1, participants listed their social groups. They also reported the size of their social network by listing the number of people they interact with daily and monthly. One value was deleted as an outlier according to the same criteria as in Study 1. Finally, participants completed a demographics survey reporting only age, gender, and relationship status.

### Results and Discussion

Overall, participants listed 3.32 (*SD* = 1.74) social groups. The number of listed social groups did not differ by gender, *t*(123) = -0.32, *p* = .747, *d* = 0.06, and did not correlate with age, *r*(125) = -.07, *p* = .467. There was also no difference between the number of social groups listed by people who were single (*n* = 44) versus in a steady relationship or married (*n* = 81), *t*(123) = -0.65, *p* = .515, *d* = -0.13.

**Fig 1:**
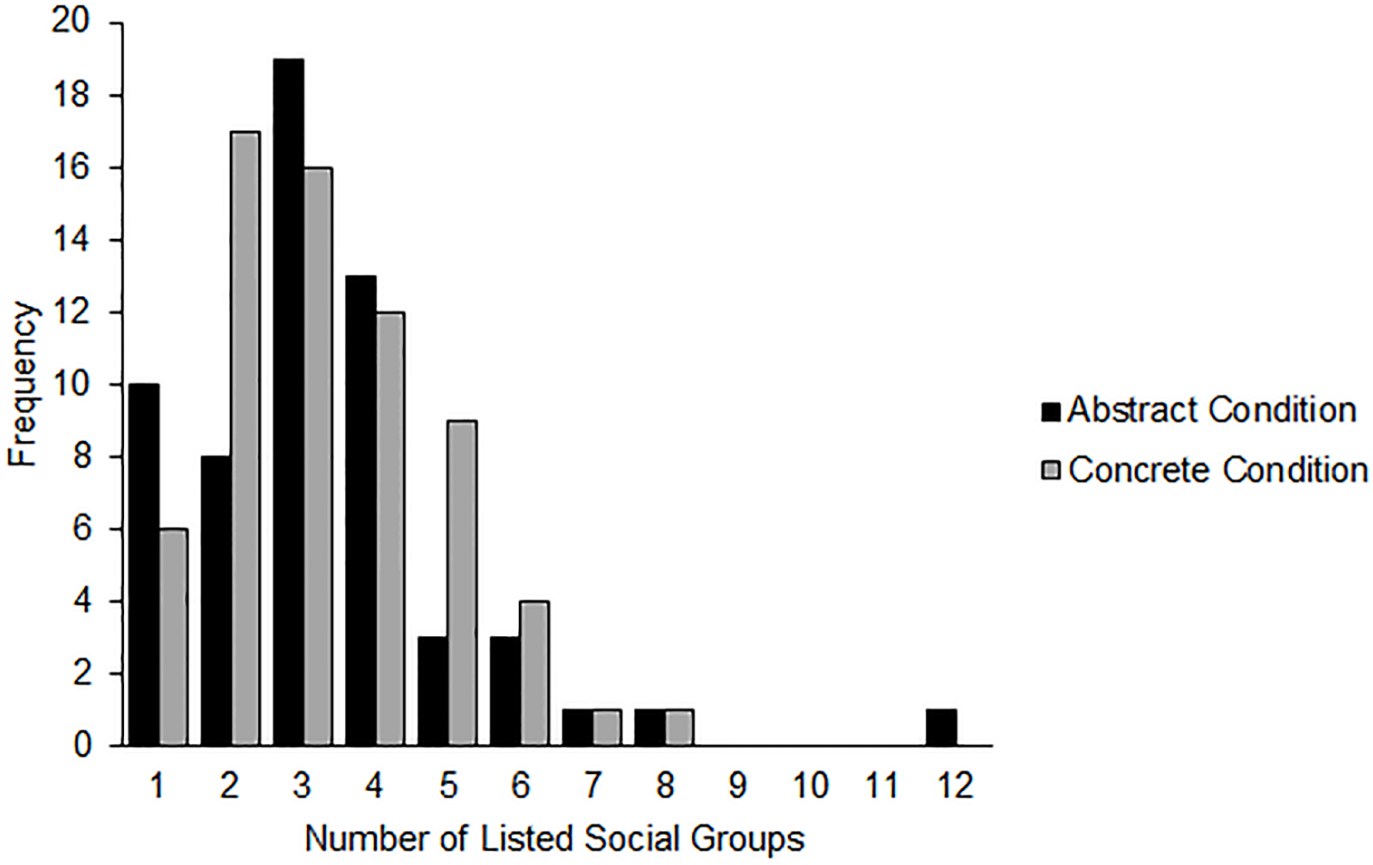
Distribution of listed social groups by construal mindset condition (Study 2).

Next, we examined the effect of condition. Preliminary analyses confirmed that the assumption of homoscedasticity was not violated, Levene's *W* = 0.082, *p* = .775 and that the mean and variance were approximately equal (Pearson dispersion = .89). We analyzed the data using a Poisson Loglinear model, examining the effect of construal mindset on the number of social groups listed. In the abstract condition, participants listed 0.99 (95% CI [0.82, 1.20]) times the number of social groups as in the concrete condition, which was not a statistically significant result, Wald χ^2^ = 0.01, *p* = .931. (see Fig 1 for the distribution of social groups by condition). Table 1 displays the means across all studies.

**Table 1:** Average number of groups generated across studies.

		Construal Mindset Condition	
		Abstract	Concrete
Study 2	Listed social groups	3.31 (1.93)	3.33 (1.56)
Study 3	Drawn social groups (circles)	4.23 (1.41)	4.48 (1.46)
	Listed social groups	4.45 (1.43)	4.64 (1.56)
Study 4	Drawn social groups (circles)	4.66 (1.83)	4.53 (1.23)
	Drawn objects groups (circles)	4.31 (1.47)	5.24 (1.89)

*Note*. The above values reflect the means, with standard deviations in parentheses.

We also investigated whether gender moderated the effect of mindset construal on the number of social groups listed. The gender by condition interaction term was not significant, Wald χ^2^ = 0.003, *p* = .956, and there was no main effect of gender, Wald χ^2^ = 0.10, *p* = .753. Additional exploratory analyses also showed that mindset condition did not affect the size of people's social network (i.e., the people they interact with daily or monthly), *p*s > .325, and that the size of the network was not related to the number of social groups, *r*s < .06.

There are a couple of explanations for the null effect of mindset condition. First, it is possible that the mindset manipulation may not have been strong enough to induce participants to adopt an abstract or concrete frame of mind. However, this manipulation [25] is well-established and has been used in almost 300 papers (2015, Google Scholar). Second, it is possible that the measure of social groups was not sensitive enough to allow participants to restructure how they thought about their various social relationships.

## Study 3

Study 3 addressed two potential explanations for the null effect of the mindset manipulation in the previous study. First, we assessed the number of social groups in a different way. Participants were asked to physically draw circles to represent their social groups. Rather than simply listing social groups, this task was expected to be a more active and cognitively demanding process in which participants thought about their social relationships before categorizing and drawing social groups to distinguish the social groups encompassing these relationships. We also asked participants to list their relationships beforehand to jog their memory and provide a more exhaustive picture of their various relationships. For validation purposes, participants also listed social groups after this drawing task. Second, we included a validation measure of the mindset manipulation. Participants completed a measure of state construal mindset [34] to examine whether the manipulation influenced their tendency to think abstractly or concretely during the experiment.

### Method

#### Participants

Eighty-one students on the campus of a Canadian University completed a short paper-and-pencil study. Participants were volunteers and received candy bars as incentive to participate. Seven participants did not follow the instructions of the construal task (e.g., did not complete survey in sequence, responses for construal task were nonsensical) and were excluded from the sample, and one participant was excluded as an outlier (number of social groups drawn was greater than three standard deviations from the mean). The final sample consisted of 73 participants (34 females, 39 males) with a mean age of 21.54 (*SD* = 6.26).

#### Procedure

Participants completed a short demographics survey (assessing only age and gender) and then thought about 30 people they knew and regularly interacted with. Space was provided for participants to record the initials for each of their various relationships. This number of relationships was chosen as prior research indicates that people have approximately 26 to 37 relationships in their social network (e.g., [13,15]).

Then, construal level was manipulated as in Study 2 [25]. Participants were randomly assigned to adopt either an abstract or a concrete mindset by a thought exercise leading them to think increasingly abstract (why they would pursue a goal) or increasingly concrete (how they would pursue a goal). Next, participants completed a drawing task. Participants read a definition of social groups which was different than the first two studies:

A social group is defined as a group of people sharing some common social relation (e.g., people you know from a specific time in your life, or people who have specific characteristics in common, or groups that have “special status” in your mind).

Next, participants read the following instructions on how to group their social contacts:

Think about the people you listed before, and use the space below to categorize these people. Draw circles to represent distinct social groups and sort these people into different circles (i.e., different social groups). Make sure that each person is sorted into a social group and that you do not include anyone in more than one social group. There is no limit to the number of people you can or cannot include in each group (i.e., you can include 1–30 people in a circle).

**Fig 2:**
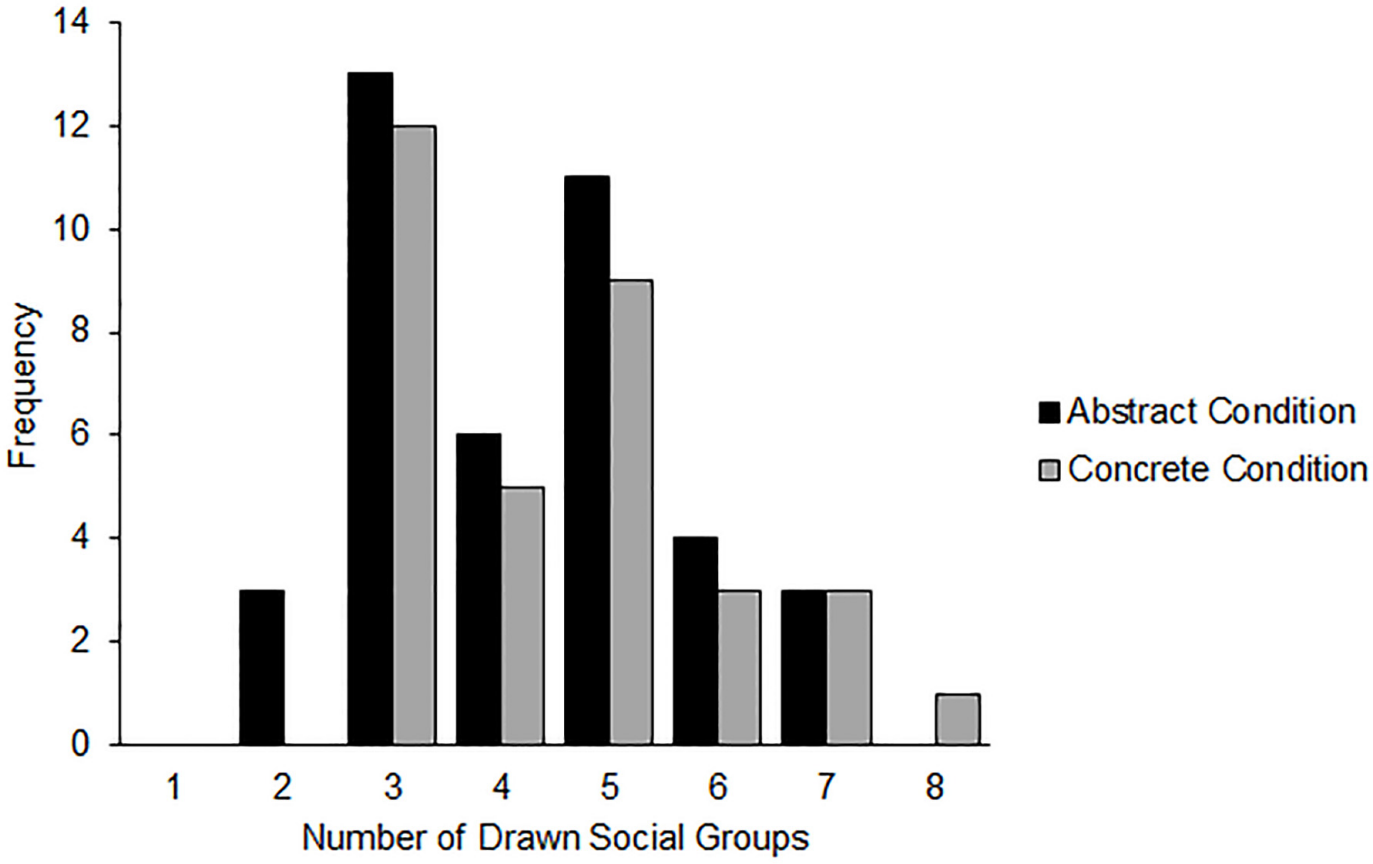
Example of how participants sorted their relationships.

Participants also indicated the name of each social group at the top of the circle. A blank page was provided for this task and an example was provided as a demonstration to participants (see Fig 2). We counted the number of circles drawn as a measure of the number of social groups. We also counted the number of people listed in each of the circles. For validation of the construal manipulation, participants also listed their social groups, in ten separate textboxes.

Finally, we assessed state construal mindset via a 13-item multiple-choice scale (a shortened version adapted from [34]). For each item, participants read a behavior and two corresponding descriptions; one description reflected an abstract mindset and the second description reflected a concrete mindset. For example, a sample behavior item included, “picking an apple”, with the corresponding responses being: “getting something to eat” (abstract response) and “pulling an apple off a branch” (concrete response). The number of abstract responses was computed to create an indicator of abstract construal mindset.

### Results and Discussion

Participants listed an average of 26.47 (*SD* = 6.73) people they regularly interact with and drew an average of 4.34 (*SD* = 1.43) distinct circles (i.e., social groups). The fewer circles they drew, the more relationships were included, on average, in each circle, *r*(70) = -.53, *p* < .001, supporting the idea that global social groups are more inclusive. Participants listed an average of 4.54 (*SD* = 1.48) social groups in the textbox measure. The number of social groups drawn was correlated with the number of social groups listed, *r*(71) = .77, *p* < .001, and did not differ significantly from the simple listing of groups, *t*(71) = -1.70, *p* = .09, *d* = 0.41.

**Fig 3:**
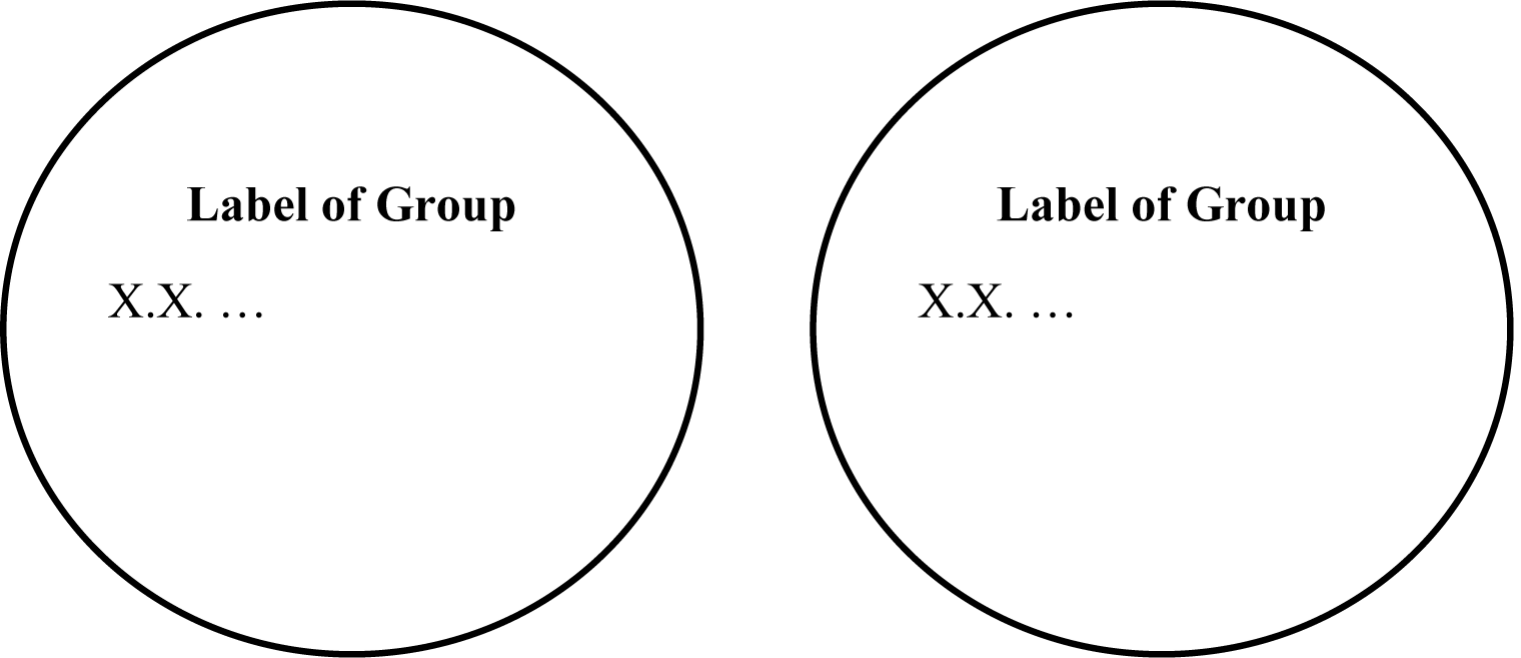
Distribution of drawn social groups by construal mindset condition (Study 3).

Next, we examined the effect of condition. Preliminary analyses confirmed that the assumption of homoscedasticity was not violated for any of the dependent variables, Levene's *Ws* < 1.31, *p*s = .255. We analyzed the data using a Poisson Loglinear model, examining the effect of construal mindset on the number of social groups drawn. In the abstract condition, participants drew 0.94 (95% CI [0.76, 1.18]) times the number of social groups as in the concrete condition, which was not a statistically significant result, Wald χ^2^ = 0.28, *p* = .596 (see Table 1 and Fig 3.). The same was true for the listed number of social groups, Wald χ^2^ = 0.14, *p* = .709.

Gender did not interact with the non-significant condition effect on drawn social groups, Wald χ^2^ = 0.42, *p* = .517, or listed social groups, Wald χ^2^ = 0.002, *p* = .963. Participants in this study were notably younger than participants in the previous two studies (early twenties instead of mid-thirties), and the type of social groups might have been influenced by that (e.g., colleagues are a less relevant social group in a student sample). However, as in previous studies, age was not correlated with the number of social groups drawn or listed, Wald χ^2^ < .77, *p*s = .381.

Finally, we examined whether the mindset manipulation affected responses on the behavioral identification form [34], that is, whether the manipulation actually shifted construal mindset. In the abstract condition, participants selected 1.21 (95% CI [1.04, 1.41]) times the number of abstract responses (*M* = 10.25, *SD* = 2.25) as in the concrete condition (*M* = 8.48, *SD* = 2.61), which was statistically significant, Wald χ^2^, = 5.94, *p* = .015 (see Table 1). Thus, the mindset manipulation effectively shifted participants' mindset, as intended, suggesting that the reason for the null effect of mindset condition on social groups was not a failure of inducing a different frame of mind. Indeed, the behavioral identification form assessing mindset was measured after the social group drawing and social group listing task, suggesting that the mindset induced by the abstract mindset manipulation was still detectably more abstract even *after* the measure of social groups.

## Study 4

The final study examined the effect of construal mindset on both structuring social groups and in structuring a control domain: household objects. As in Studies 2 and 3, we expected that mindset would not have an effect on the number of social groups participants chose to represent their social network. However, when comparing this negative result with a domain in which mindset does change mental representation, we expected that mindset would have an effect on the number of object categories participants would chose to categorize objects into.

This control domain (categorizing objects) is closely modeled on past research [33] that showed a difference in level of categorization depending on construal mindset. Liberman and colleagues [33] randomly assigned participants to think about a hypothetical scenario (e.g., going camping, moving out, going on a NYC visit) while adopting either a concrete or abstract mindset. Participants were presented with a list of 38 objects related to the particular scenario and asked to sort each item based on what items belong with each other. Participants drew circles around each set of objects to classify them into distinct groups. The researchers counted the number of groups drawn and found that participants in an abstract mindset drew fewer groups to classify the objects than participants in a concrete mindset. Thus, we expect that a measure of grouping of objects–but not of social groups–will show differences in categorization levels depending on participants' frame of mind. We expect that participants will form more abstract, global groups when adopting an abstract mindset for the control domain (categorizing objects) but not for the social domain (categorizing relationships).

### Method

#### Participants

For practical reasons we did not collect all responses in individual lab sessions, but collected as many responses as we could over two semesters in the lab and also collected online responses for the identical questionnaire, from the identical pool of participants (undergraduate students at a Canadian University). In sum, 23 participants completed this study in the lab and 191 participants completed the study online. In the online sample, 17 participants did not finish the survey (five participants did not complete the manipulation, 12 participants did not complete the main dependent measure) and were excluded.

In the online questionnaire, we had to trust that participants would actually complete the social groups drawing task on their own (where in the lab we could observe them do it). To determine whether participants were likely to have actually done the drawing task, they were timed. Participants in the lab spent between 218.40 and 670.38 seconds on the drawing task (*M* = 434.45 seconds, *SD* = 111.84). As per guidelines for dealing with outliers in data [35], and consistent with our outlier criteria in Studies 1 through 3, we excluded participants who took less than three standard deviations below this average completion time because we suspected these participants did not really do the drawing task. Exclusions did not differ across domain (relationships vs. objects), χ^2^(*df* = 1, *N* = 197) = 0.49, *p* = .486. The final sample consisted of 147 participants (116 female, 31 male) with a mean age of 20.06 (*SD* = 3.69).

### Procedure

Participants completed a short demographics section assessing age and gender and were randomly assigned to either the categorizing relationships or categorizing objects condition. As in Study 3, participants in the relationships condition listed 30 people they knew and regularly interacted with, whereas participants in the objects condition listed 30 household objects that they own and regularly use. Next, participants were assigned to either the abstract or concrete mindset condition, as in previous studies [25,32].

Participants completed a drawing task where they were asked to sort the 30 relationships (or 30 household objects) that they had listed into circles, representing social groups. The drawing task instructions for the social groups condition and definition of social groups were identical to that of Study 3. Parallel instructions were given to participants in the objects condition: participants were told that household objects were goods or products that were found in one’s home which can be grouped into a variety of different groups based on certain characteristics. Participants who completed the study online were told to complete this task on a sheet of paper and then to indicate the total number of groups (i.e., circles) they drew as well as the number of people or objects that were drawn in each circle. To assess the extent of participation in this task, we tracked the total time spent on this page. The variable denoting whether the study had been conducted in lab or online was not a significant covariate or moderator of the results reported below and will not be discussed further. Participants in the objects condition and the relationships condition did not differ in age, *t*(145) = 0.70, *p* = .486, but there were proportionally more female participants in the objects condition, *χ*^2^ = 4.13, *p* = .042.

Next, participants rated how difficult it was to sort their relationships or objects into distinct groups on a 7-point scale from 1 (*not at all difficult*) to 7 (*very difficult*). On a scale from 1 (*never*) to 7 (*always*), participants also rated how often they think about their various relationships (or household objects) in terms of specific groups. Finally, participants were asked to respond either “yes” or “no” to whether they: found it easy to group their relationships (or objects) into distinct groups, preferred to have less specific instructions for grouping, and preferred to have been able to group into overlapping or nested groups.

### Results and Discussion

The average number of relationships listed was 28.66 (*SD* = 4.35) and the average number of household objects listed was 29.74 (*SD* = 1.64), *t*(145) = -2.01, *p* = .046, *d* = 0.33. Participants listed an average of 4.59 (*SD* = 1.55) groups in the relationships condition and 4.76 (*SD* = 1.74) groups in the household objects condition, *t*(145) = -0.63, *p* = .530, *d* = -0.10. Thus, the number of social groups drawn was comparable to the number of social groups reported in Study 3 (similar population and same measure).

**Fig 4:**
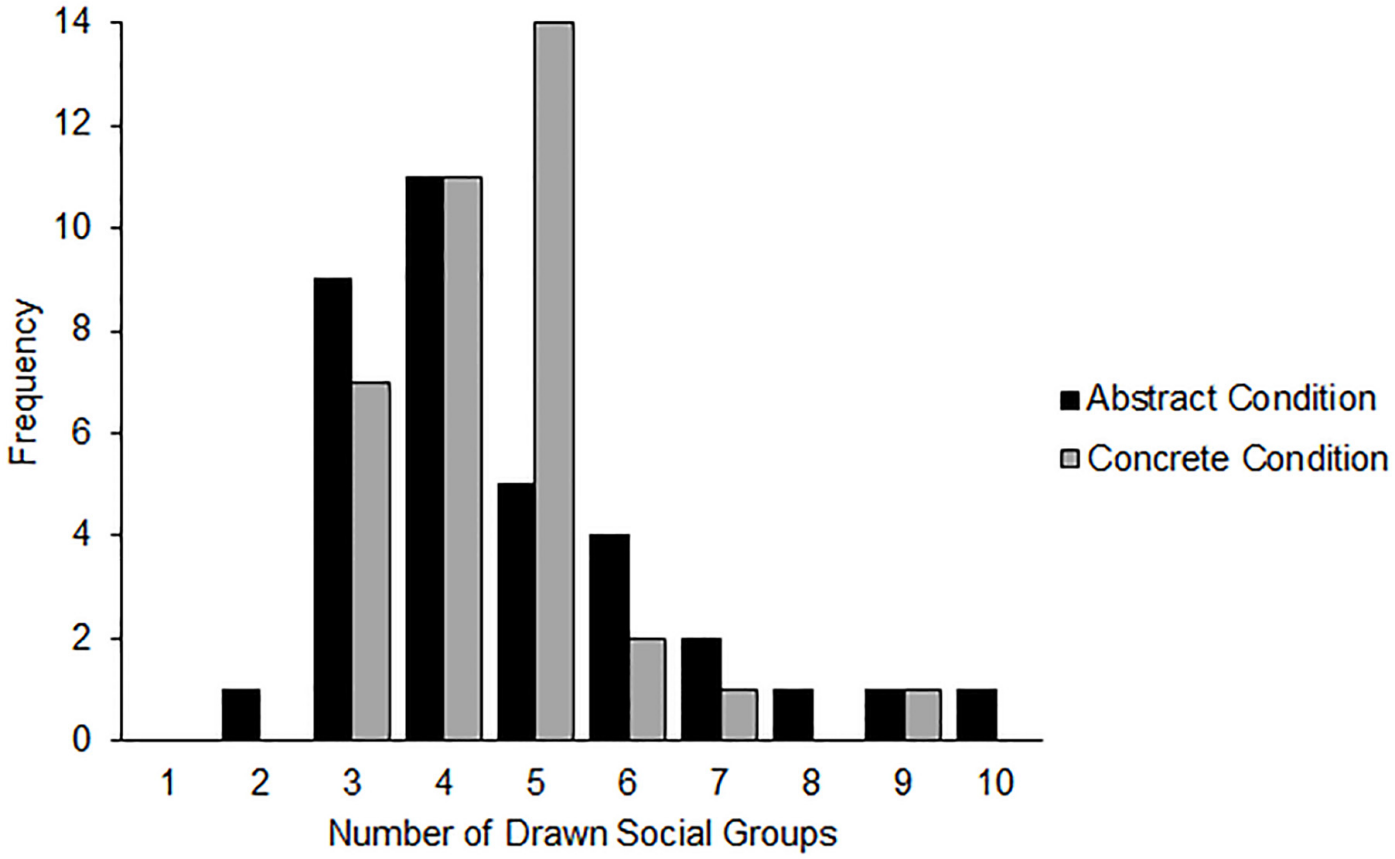
Distribution of drawn social groups by construal mindset condition (Study 4).

**Fig 5:**
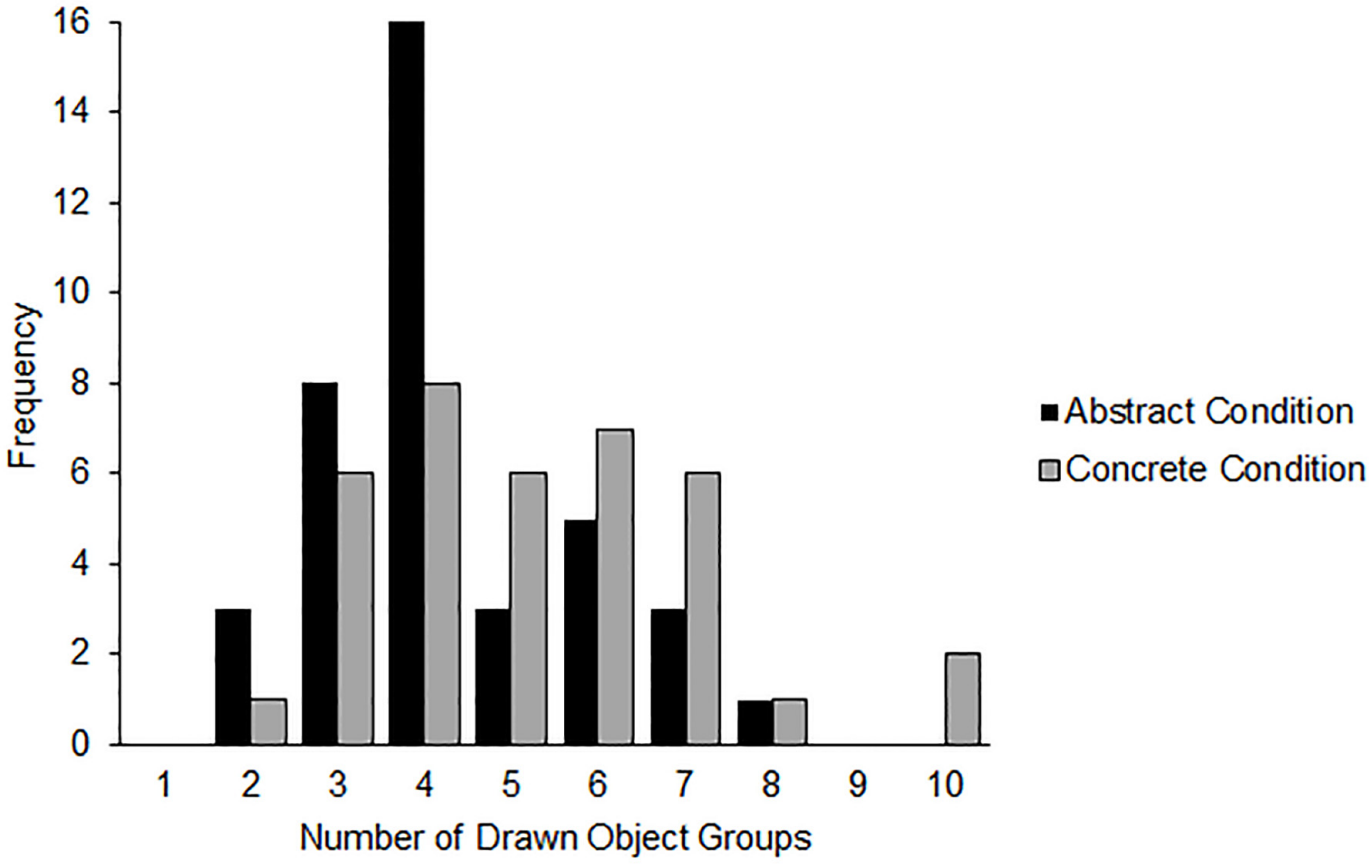
Distribution of drawn objects groups by construal mindset condition (Study 4).

Next, we examined the effect of the mindset condition on categorizing objects and relationships. Preliminary analyses confirmed that variances were homogenous across abstract and concrete mindset conditions, Levene's *W* = 2.57, *p* = .111, and across the two domains, Levene's *W* = 0.18, *p* = .672. We analyzed the data using a Poisson Loglinear model, examining the effect of construal mindset on the number of social groups drawn. When categorizing objects, participants in the abstract condition drew .82 (95% CI [0.67, 1.01]) times the number of groups as those in the concrete condition, which was a marginally statistically significant result, Wald χ^2^ = 3.47, *p* = .062. However, when categorizing relationships, participants in the abstract condition drew 1.03 (95% CI [0.83, 1.28]) times the number of social groups as those in the concrete condition, which was not a statistically significant difference, Wald χ^2^ = 0.07, *p* = .799. The distribution of groups by construal mindset are displayed for the relationships condition (Fig 4) and objects condition (Fig 5). In sum, this final study suggests that the abstraction of thought induced by the construal level mindset manipulation used did indeed shift the grouping and structuring of some stimuli (household objects)–however, as in the previous studies, it did not shift the structure of social relationships.

Gender did not interact with mindset condition in the relationships domain, Wald χ^2^ = 0.09, *p* = .771, or in the objects domain, Wald χ^2^ = 0.04, *p* = .837. Age was again not correlated to the number of groups drawn for all conditions, *r*s .03 to -.25, *p*s < .142.

Exploratory analyses examined whether people believe they naturally think about their relationships versus household objects in terms of groups. There was no statistical difference in participants’ rating of difficulty for the objects task (*M* = 3.81, *SD* = 1.69) versus the relationships sorting task (*M* = 3.59, *SD* = 1.72), *t*(141) = -0.78, *p* = .436, *d* = 0.13. However, participants in the relationships condition reported that they thought about their relationships in terms of specific groups (*M* = 3.60, *SD* = 1.48) more often than participants reported thinking about their household objects in terms of groups (*M* = 2.79, *SD* = 1.53), *t*(141) = 3.20, *p* = .002, *d* = 0.54. Although preliminary, this might suggest that people tend to spontaneously categorize their relationships into social groups, which predisposes them to construct these groups in a specific way–whereas household objects might not be spontaneously grouped and changes in cognitive mindset can more easily shift their categorization.

## General Discussion

We expected that the level of abstraction in people's thoughts would affect the level of abstraction in how they organized their social relationships into social groups. However, three studies showed no effect of construal level mindset on the grouping of social relationships. The construal level manipulation [25] is a well-established method to shift the level of abstraction in people's mindsets. Furthermore, Study 3 provided evidence that this manipulation did indeed shift participants' thoughts to be more abstract, and Study 4 provided evidence that this manipulation did affect the categorization of objects (control condition) in a way that was consistent with expectations (abstract thoughts led to more abstract, global categories). We conclude that the null effect of construal mindset on social group structure is meaningful rather than incidental. Social relationships are mentally grouped in a way that is independent from the level of mental abstraction.

### Measuring Social Grouping

Our measures of social groups were designed to most closely resemble the way social groups are presented in the social media people regularly interact with (Facebook, Google+) and were open-ended in order to allow for changes in group structuring. Indeed, there were considerable differences in the number of social groups between participants, if none between conditions. Notably, we measured the grouping of household objects in an identical way (Study 4), and there the mindset manipulation had the expected effect. Thus we would argue that the null effect is not attributable to the way social group categorization was measured. This being said, there may be a number of other ways to assess the structure of people's social network. For instance, future research may use cluster analysis [36], as a more indirect way to assess the categorization of social groups instead of relying on self-report measures.

Notably, participants in Study 3 and 4 listed a restricted number of relationships before the construal manipulation, and then sorted these people into social groups. This procedure was used to ensure that the number of social relationships was equal across construal manipulations. However, it is possible that when listing relationships, participants may have thought of one person (e.g., someone from their hockey team) and then been reminded of other people who have similar attributes or are from a similar context (e.g., other hockey teammates). Thus, the salience of one person and similar others thereafter may have artificially induced participants to think about their relationships in terms of social groups even before participants were explicitly asked to group their relationships. It is also possible that limiting the number of social relationships to 30 may have affected participants' structures of social groups. Future research may examine a complete mapping of the social network by asking participants to list an exhaustive list of social contacts before generating social groups.

It should be acknowledged that everyone embeds and immerses themselves into their social networks differently. The identification of one’s social network may not always be apparent and requires reflection by the individual [37]. And although thinking in terms of social groups might be an artificial process for some, people are now connected to social media more than ever before–a place where grouping of relationships is frequently imposed.

### Implications of Social Grouping

The categorization and structure of social relationships is relevant within a variety of day-to-day occurrences. First, people's identity may be linked to the type of social groups they belong to [3]–reframing one's social network in different groups (e.g., higher-order groups such as "family" versus lower-order groups differentiating "in-laws" from "household family") may affect the importance of these groups. Furthermore, the valence and emotional meaning of the groups may shift alongside the relationships that are included in them.

Second, relationships require effort to be maintained [38], yet resources need to be managed across a number of important social relationships. People might make trade-offs when determining pro-relational maintenance behaviors. For example, after having watched a movie with one friend, proofread a different friend's term paper, and send a card for yet another friend's birthday, an individual may be less likely to agree to take care of a friend's children that same evening (i.e., same social group) but might feel differently if her sister asks her to babysit (i.e., different social group). How people allocate their effort might be determined by the social group membership of the help recipient.

Third, the grouping of social relationships could affect how similar or closely connected one group is to another. For instance, work-family conflict has been linked to a lack of boundaries between work and family domains–where individuals have a hard time separating one from the other [5].

### Why does mental abstraction not affect social grouping?

Why does the way people structure their relationships in social groups remain unaffected by shifts in the abstraction of thoughts (i.e., construal mindset)? Construal mindset is a powerful influence on how we think about the world (for a review, see [22,23]), though why did it not affect thoughts on relationship grouping? One reason for the null effect of construal mindset in this particular domain might be the emotional connection between participants and their self-reported social groups. Thinking about how social relationships "fit" together is a more emotional experience than considering how household objects "fit" together. In other words, a peeling knife might sometimes be thought of as cutlery and sometimes as a tool, but someone's favorite aunt is not easily thought of as belonging to the same social group as disliked family members. Motivation to accurately represent emotional connections and emotional variability in the objects being sorted (i.e., how to classify the favorite aunt may be an emotional question whereas how to classify the peeling knife is not) might make the mental structure of social groups immune to cognitive shifts in construal level mindset. To examine this possibility, future research could ask participants to generate social groups in circumstances where the emotional connectivity is reduced–for example, generating groups for someone else’s network of relationships or for people who they do not intimately know (e.g., political leaders, celebrities).

Another possibility is that people are more likely to spontaneously categorize their social relationships into distinct groups–more so than they are to spontaneously organize other aspects, which may have a less embedded structure and be easier to shift. Study 4 offered some preliminary evidence for the idea that people find it more natural to think in terms of groups for their relationships than for everyday objects.

## Conclusions

Across three studies, mental abstraction did not affect the degree of abstraction when it came to categorizing relationships into social groups. Mental abstraction affects a variety of thoughts, feelings, and behaviors in everyday life [29–33], and as such may be seen as one of the most powerful aspects of cognition coloring how we see the world. However, mental abstraction did not shift social network organization, implying that how people organize their relationships seems to be determined by factors other than moment-to-moment shifts in cognition. How social groups are structured may be too meaningful to people to lightly shift from moment-to-moment and might instead be determined by deep-seated principles common across people or by stable individual differences.
