# Correction: Thinking Big or Small: Does Mental Abstraction Affect Social Network Organization?

**DOI:** 10.1371/journal.pone.0160047

**Published:** 2016-07-20

**Authors:** Chantal Bacev-Giles, Johanna Peetz

The images for Figs 2 and 3 are incorrectly switched. The image that appears as Fig 2 should be Fig 3, and the image that appears as Fig 3 should be Fig 2. The figure captions appear in the correct order. See the corrected Figures below.

**Fig 2:**
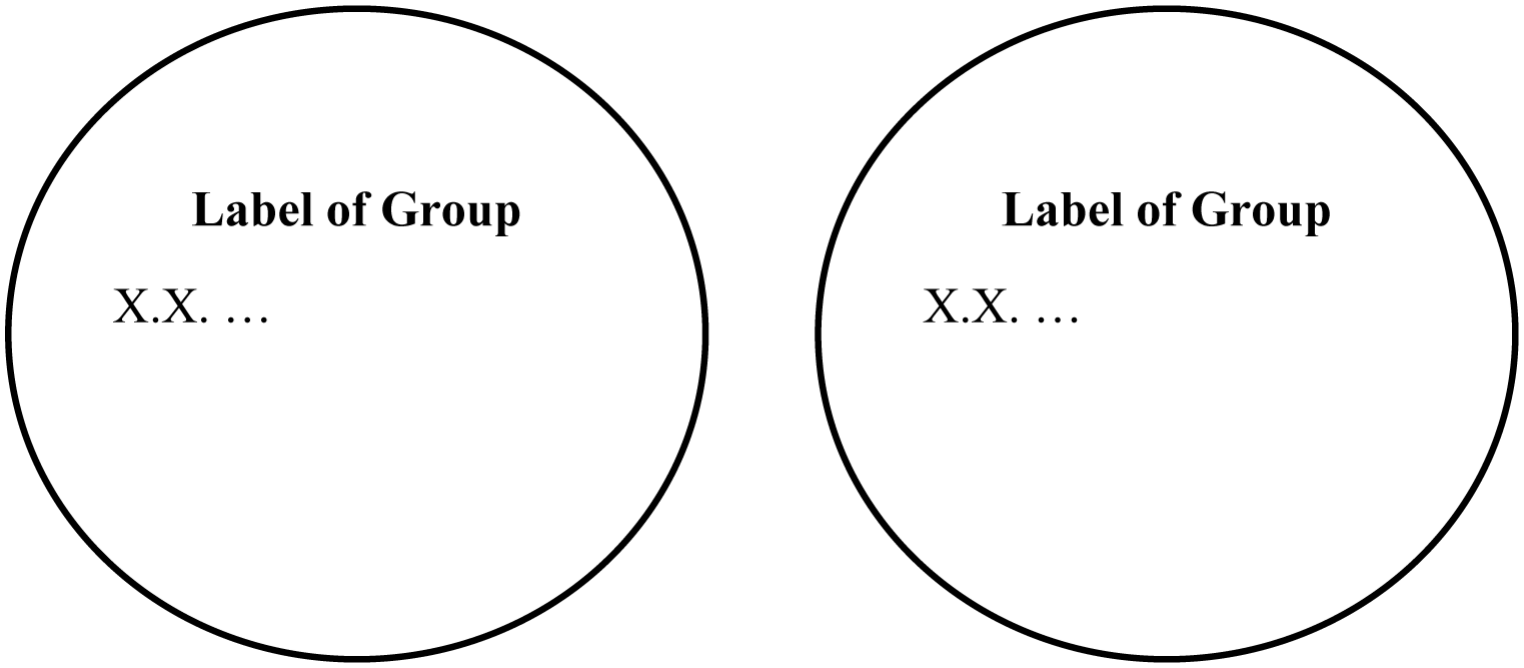
Example of how participants sorted their relationships.

**Fig 3:**
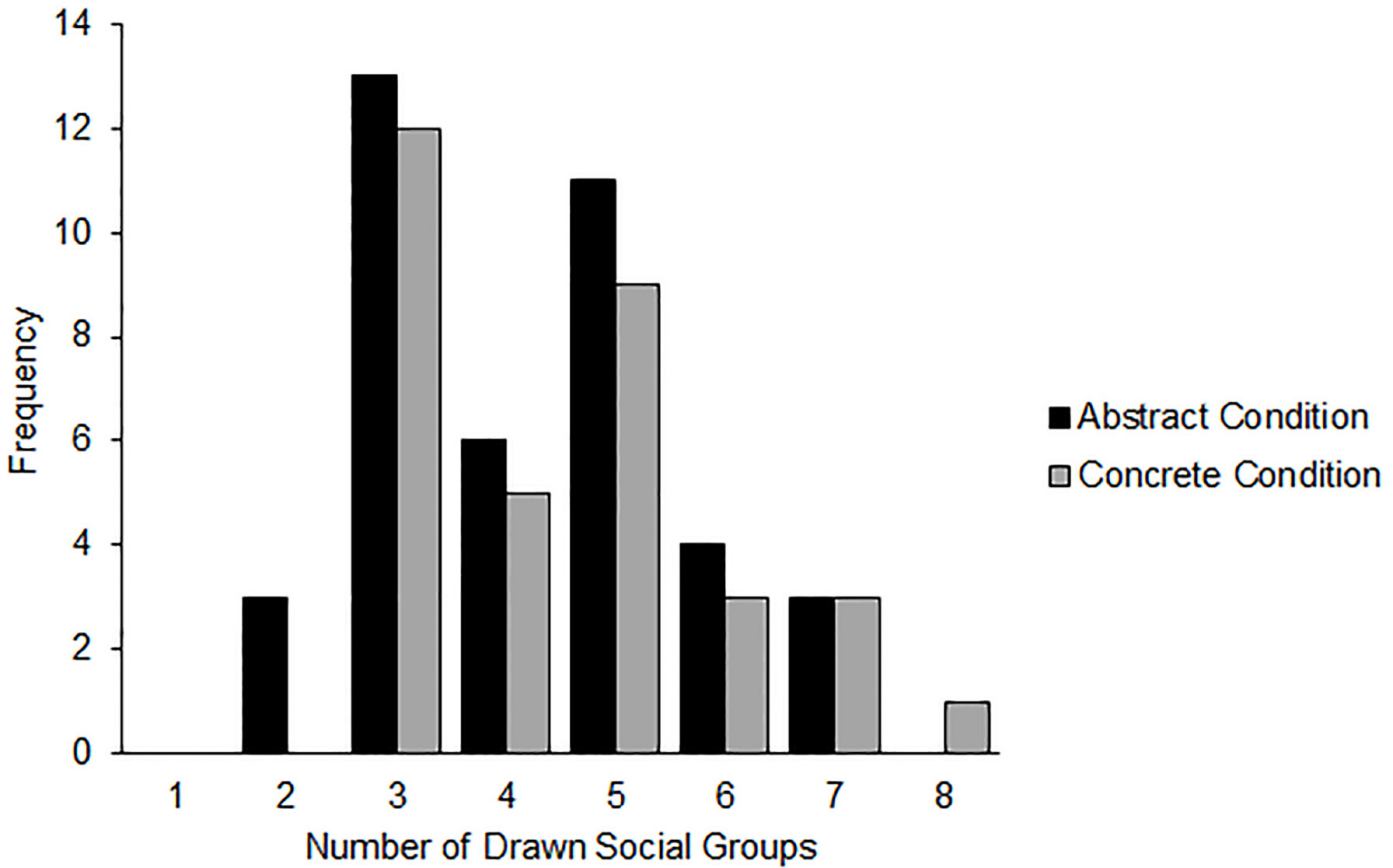
Distribution of drawn social groups by construal mindset condition (Study 3).
